# Knowledge and practices related to antibiotic use among women in Malang, Indonesia

**DOI:** 10.3389/fphar.2022.1019303

**Published:** 2022-10-24

**Authors:** Sendi Lia Yunita, Hui-Wen Yang, Yi-Chun Chen, Li-Ting Kao, Yi-Zi Lu, Yuan-Liang Wen, Sheng-Yin To, Ya-Li Huang

**Affiliations:** ^1^ Pharmacy Department, Faculty of Health Science, University of Muhammadiyah Malang, Malang, Indonesia; ^2^ Graduate Institute of Life Sciences, National Defense Medical Center, Taipei, Taiwan; ^3^ School of Public Health, National Defense Medical Center, Taipei, Taiwan; ^4^ School of Nutrition and Health Sciences, College of Nutrition, Taipei Medical University, Taipei, Taiwan; ^5^ School of Pharmacy, National Defense Medical Center, Taipei, Taiwan; ^6^ Department of Pharmacy Practice, Tri-Service General Hospital, Taipei, Taiwan; ^7^ School of Public Health, College of Public Health, Taipei Medical University, Taipei, Taiwan; ^8^ Department of Public Health, School of Medicine, College of Medicine, Taipei Medical University, Taipei, Taiwan

**Keywords:** antibiotic, knowledge, self-medication, practices, women, Indonesia

## Abstract

**Background:** Antimicrobial resistance is a public health problem that threatens the efficacy of antibiotics. Incorrect knowledge of antibiotics may lead to their inappropriate use, hinder their effectiveness, and cause antibiotic resistance. Population-based educational campaigns have been found to have either mixed or no effect on improving knowledge and appropriate antibiotic practices, suggesting the need for more targeted approaches in tailoring education for specific subpopulations. Women are the primary caregivers of their families and are more willing to contact healthcare providers. They had greater knowledge of antibiotics and better adherence to the completion of the antibiotic regimen. Therefore, they are suitable for prioritization in a campaign program.

**Objective:** This study examined the knowledge and practices of female visitors to health centers in Malang, Indonesia with respect to antibiotic use.

**Methods:** This cross-sectional study was conducted in Malang, Indonesia, in July and August 2018. Data were collected from 677 women. Multivariate logistic regression was performed to identify the potential factors associated with antibiotic knowledge, self-medication, and completion of antibiotic regimens.

**Results:** Overall, 82.7% of respondents were aware that antibiotics are used against bacteria, while 38.4% reported self-medication with antibiotics and 51.7% reported completing antibiotic regimens. Women with higher education, previous antibiotic use experience, and very easy accessibility to primary doctors were more likely to have high antibiotic knowledge than those with primary education, no antibiotic use in the previous year, and easy/other level of accessibility to primary doctors. Subjects residing in urban areas and with less accessibility to primary doctors were more likely to self-medicate with antibiotics. Additionally, the completion of antibiotic regimens was positively associated with access to a primary care doctor and high antibiotic knowledge.

**Conclusion:** IF Policymakers tend to reduce inappropriate antibiotic use among women. Priority advocates are recommended for urban residents who have experiences of antibiotic use in the previous year. It is therefore important to increase their awareness, particularly regarding diseases against which antibiotics are effective, and activities such as unnecessary use of antibiotics in healthy animals, which may affect their overall effectiveness among humans. More communication channels should be included in the overall scheme to improve the public awareness and accessibility of health professionals.

## 1 Introduction

Antibiotic resistance (ABR) is a global threat that leads to treatment failure, longer hospital stays, increased cost of care, morbidity, and mortality (Friedman et al., 2016; Yau et al., 2021). The studies by Alnemri et al. (2016); Karuniawati et al. (2020) proposed that among the causes of ABR, misuse and overuse of antibiotics directly affect the development of ABR due to lack of knowledge, careless attitudes, and incorrect beliefs about antibiotics among the public. Antibiotic use without a prescription, use of leftover antibiotics, and discontinuing use without consulting a health professional are common behaviors among users and may be associated with the reported spread of antibiotic resistance (Al Omari et al., 2019). Therefore, considering the public health importance of this issue, the World Health Organization (WHO) recommends that antibiotics should only be used when prescribed by a certified health professional, discourages the sharing and use of leftover antibiotics, and encourages regimen completion even when symptoms disappear.

Inappropriate antibiotic use is reportedly higher in low- and middle-income countries (Nepal and Bhatta, 2018). A study found that approximately 69% of study participants were taking non-prescribed antibiotics or using leftover medications (Belkina et al., 2014). Gender, educational level, living area, and monthly income have been reported to be associated with knowledge of antibiotics. A study conducted in Indonesia indicated that female subjects with higher-level education, higher income, and living in an urban area have better knowledge of antibiotic use (Karuniawati et al., 2021). Regarding self-medication, studies conducted in developing countries such as Sudan and Nigeria indicated that younger age, female sex, lower income, and higher level of education were significantly associated with an increased risk of self-medication with antibiotics (Awad et al., 2005; Osemene and Lamikanra, 2012).

Several studies have attempted to determine the factors that may affect these key points to prevent ABR problems. However, population-based educational campaigns were found to have either mixed or no effects on improving knowledge and appropriate antibiotic practices, suggesting the need for more targeted approaches in tailoring education for specific subpopulations (Lim et al., 2019). Previous studies have showed that women were more motivated to visit health care facilities than men, regardless of acute or chronic conditions (Balbay et al., 2005; Babwah et al., 2006; Fatokun, 2014). Additionally, several studies indicated that women have more frequent contact with healthcare providers over their life course than men, which may be due to female reproductive factors such as pregnancy, birth, and menopause, as well as experience with antibiotics while caring for children and other family members (Baker et al., 2007; Maume, 2008; Thompson et al., 2016; Burnside et al., 2018; Shebehe et al., 2021). Furthermore, one study provided evidence that most family caregivers were female and around the reproductive age (Togoobaatar et al., 2010). Women were the primary caregivers of the family. They had greater knowledge of antibiotics and better adherence to the completion of the antibiotic regimen (Fatokun, 2014; Karuniawati et al., 2021; Endashaw Hareru et al., 2022). However, these patients are prone to self-medication (Awad et al., 2005; Aditya and Rattan, 2013; Israel et al., 2015; Ramay et al., 2015; Kurniawan et al., 2017; Torres et al., 2019).

The emergence of antibiotic resistance and widespread antibiotic misuse warrants research on knowledge of antibiotic usage and related practices. In Indonesia, the purchasing of antibiotics is allowed only based on prescription. The exception is for antibiotics that are included in Obat Wajib Apotek (OWA) (the list of medicines that can be given by a pharmacist without prescription, such as gentamicin for topical usage), which are allowed to be used for self-medication. A common dispensing pattern was the direct request of antibiotics by clients, who walked into pharmacies or drug stores and asked for antibiotics without prescription, either by their generic/brand name or by showing an empty package or sample ( Ferdiana et al., 2021 ). There is widespread existence of pharmacies and kiosks, which subsequently improve access to medicines and facilitate self-medication (André et al., 2010). Most studies concerning knowledge of antibiotic usage and associated behaviors (i.e., self-medication and completion of antibiotic regimens) in Indonesia have focused on the general population. To our best knowledge, relevant information regarding women is limited.

Therefore, determining the potential factors that may affect women’s attitudes or behaviors toward antibiotics is an important task to address the research gap and for future policy planning. This study aimed to assess the knowledge levels regarding antibiotics and factors associated with antibiotic knowledge level, completion of antibiotic regimens, and practice of self-medication for women in Malang, Indonesia.

## 2 Methods

### 2.1 Study area and design

The study was conducted in Malang, Indonesia. Malang is part of East Java Province, which lies on Java Island. The population of Malang is the second highest in East Java, with approximately 3.35 million inhabitants. This cross-sectional survey was conducted in July and August 2018 at four primary healthcare centers called puskesmas. Prior to the study, primary healthcare centers in Malang were contacted to inquire about their availability for participation and to assist with data collection. Finally, four primary healthcare centers, two located in the city center and two in rural areas, were selected for their locations and willingness to collaborate. Convenience sampling was used in this study. Female visitors aged 18–49 years and who were able to communicate (read, write, listen, and speak) in Bahasa Indonesia (i.e., the official language of the Republic of Indonesia) were invited to participate in this study. If both patients and companions were present, only one participant was asked to participate. In total, 677 visitors were contacted and 583 completed the questionnaire.

After conducting a review of comparable studies, we developed a structured questionnaire to collect data (André et al., 2010; Hoffmann et al., 2013; Bert et al., 2016; Carter et al., 2016). The questionnaire included questions related to sociodemographic factors, health status, antibiotic use, sources of information, use of other services, and knowledge of antibiotic use.

We developed the questionnaire in English. Two Indonesian master students from Taipei Medical University who were familiar with Bahasa Indonesia were invited to translate and back-translate the questionnaire before and after the pretesting to ensure consistency with the original questions and contextual applicability in terms of cultural differences. The developed questionnaire was pretested once by 10 local residents. The pretesting results indicated that the questionnaire is clearly understandable, following which we used it for data collection.

Trained assistants collected data through standardized interviews. They explained the aim and content of this study as well as informed consent to potential respondents. If the respondents were unable to read, the questionnaire was read to them.

### 2.2 Measurements

Knowledge of antibiotics was assessed using the 11 questions listed in Table 2, including knowledge regarding their appropriate use, effectiveness, and side effects. Respondents were asked whether they “agree,” “disagree,” or “do not know.” The knowledge score was calculated as the sum of correct responses to the 11 questions. Thereafter, the median was used as the cutoff point to divide respondents into “high” and “low” levels of antibiotic knowledge (You et al., 2008).

Antibiotic usage behaviors were assessed using the following two questions: for antibiotic self-medication, “Do you purchase antibiotics without a medical prescription?” (yes/no), while for the completion of antibiotic regimens, “Do you always complete the course of treatment with antibiotics even if you feel better?” (yes/no).

Other independent factors included sociodemographic and health-related variables. The participants’ sociodemographic characteristics included residence (rural or urban), age (18–29, 30–39, or 40–49 years), marital status, and educational level. Marital status was a dichotomous variable categorized as single (including divorced and widowed) or married. Educational level was categorized as low (junior high school or less), middle (senior high school), or high (college or above).

Health-related variables included having health insurance (yes or no), previous use of antibiotics in the past year (yes or no), self-rated health (very good, good, fair, or poor), access to a primary care doctor (very easy, easy, fair, or with some difficulty), and source of antibiotic information. The source of information indicated whether respondents had received antibiotic information from either of two professional sources—a doctor or pharmacist. Those who had received antibiotic information from both a doctor and pharmacist, either a doctor or pharmacist, and none were respectively coded as “2,” “1,” and “0.”

### 2.3 Data analysis

The completed questionnaire was checked and coded before being entered into Excel. The data were digitally stored and analyzed using SPSS version 18.0 (SPSS, Chicago, IL, United States). Descriptive data are presented as frequency and percentage or mean and standard deviation (SD), as appropriate. Bivariate associations between the independent and dependent variables were assessed using the chi-squared test. Multivariate logistic regression models were used to assess factors independently associated with antibiotic knowledge, antibiotic self-medication, and completion of antibiotic courses. The strength of the association was reported using adjusted odds ratio (aOR) and 95% confidence interval (CI).

### 2.4 Ethical considerations

Before conducting the study, an informed consent form and other related documents were reviewed and approved by the ethical clearance committee of the University of Muhammadiyah Malang (reference no.: E.5.a/226b/KEPK-UMM/VII/2018). Additionally, the written informed consent form was obtained from the participants before they responded to the questionnaire.

## 3 Results

Table 1 lists the characteristics of the study participants. More than two-thirds (71.0%) of the participants were from urban areas, and 53.0% were aged 18–29 years. A high proportion of respondents were married (56.3%), had attained tertiary education (41.5%), had health insurance coverage (65.7%), reported not using antibiotics in the past year (51.3%), rated their health as being good (69.5%), reported having easy access to a primary care doctor (61.4%), and had obtained information about antibiotics from either a doctor or pharmacist (43.7%).

**TABLE 1:** Characteristics of the study population (*n* = 583).

Variable	*n*	%
Residence		
Rural	169	29.0
Urban	414	71.0
Age (years)		
18–29	309	53.0
30–39	156	26.8
40–49	118	20.2
Marital status		
Single	255	43.7
Married	328	56.3
Educational level		
Primary	110	18.9
Secondary	231	39.6
Tertiary	242	41.5
Have health insurance		
Yes	383	65.7
No	200	34.3
Used antibiotics in the past year		
Yes	284	48.7
No	299	51.3
Self-rated health		
Fair or poor	94	16.1
Good	405	69.5
Very good	84	14.4
Access to a primary care doctor		
Very easy	140	24.0
Easy	358	61.4
Others	85	14.6
Information source about antibiotics		
0	101	17.3
1	255	43.7
2	227	38.9

SD, standard deviation.

In total, data from 583 participants were analyzed, of which 33.5% reported self-medication with antibiotics and 52.8% reported completing antibiotic regimens. Information on the knowledge of antibiotics among the respondents is summarized in Table 2. Approximately 82.7% of respondents correctly stated that antibiotics are medicines used to kill bacteria, and 80.8% accurately identified amoxicillin/ampicillin as examples of antibiotics. Additionally, 68.6% of the respondents correctly mentioned that antibiotics had to be taken completely, even if the symptoms disappeared, while over half of the respondents (55.6%) accurately said bacteria can become resistant to antibiotics. Among others, items with particularly low knowledge (i.e., with < 50% of participants giving the correct response) included “antibiotics are effective against viruses” (only 12.5% correctly dismissed this statement), “antibiotics work on most common colds” (only 22.3% correctly dismissed this statement), and “antibiotics used on animals can reduce effectiveness of antibiotics among humans” (only 17.7% correctly agreed with this statement).

**TABLE 2:** Percentages of respondents with correct knowledge.

Item no.	Questions regarding knowledge on antibiotics	% Correct response
1	Antibiotics are medicines that are used to kill bacteria.	82.7
2	Antibiotics can kill all bacteria in the body.	28.3
3	Antibiotics are effective against viruses.	12.5
4	Amoxicillin/Ampicillin are examples of antibiotics.	80.8
5	Antibiotics work on most common colds.	22.3
6	Antibiotics have to be taken completely even if the symptoms are gone.	68.6
7	Antibiotics used on animals can reduce the effectiveness of antibiotics among humans.	17.7
8	Antibiotics must be taken as soon as a person has a fever.	27.8
9	Bacteria can be resistant to antibiotics.	55.6
10	People can be allergic to antibiotics, and this can lead to death.	42.9
11	Antibiotics can prevent any illness from becoming worse.	24.9

Table 3 compares the knowledge levels and practices regarding antibiotics among female visitors with different characteristics. The factors significantly associated with a higher level of knowledge were urban residence, higher educational level, prior experience in using antibiotics, easier access to primary care, and having professional sources of information. Bivariate analyses revealed that self-medication with antibiotics was positively associated with living in an urban area, being younger, single, and having previous experience of antibiotic use. Similarly, taking complete courses of antibiotics was positively associated with having used antibiotics in the past year, having easy access to a primary care doctor, and having a high antibiotic knowledge level.

**TABLE 3:** Distribution of participants’ characteristics by knowledge and antibiotic usage behaviors.

Variable	Knowledge		Self-medication		Completing antibiotic regimens	
	Low *n* = 269	High *n* = 314	Yes (%) *n* = 195	No (%) *n* = 388	Yes (%) *n* = 308	No (%) *n* = 275
Residence						
Rural	101 (59.8)	68 (40.2)***	28 (16.6)	141 (83.4)***	80 (47.3)	89 (52.7)
Urban	168 (40.6)	246 (59.4)	167 (40.3)	247 (59.7)	228 (55.1)	186 (44.9)
Age (years)						
18–29	137 (44.3)	172 (55.7)	118 (38.2)	191 (61.8)*	155 (50.2)	154 (49.8)
30–39	77 (49.4)	79 (50.6)	48 (30.8)	108 (69.2)	90 (57.7)	66 (42.3)
40–49	55 (46.6)	63 (53.4)	29 (24.6)	89 (75.4)	63 (53.4)	55 (46.6)
Marital status						
Single	110 (43.1)	145 (56.9)	107 (42.0)	148 (58.0)***	124 (48.6)	131 (51.4)
Married	159 (48.5)	169 (51.5)	88 (26.8)	240 (73.2)	184 (56.1)	144 (43.9)
Educational level						
Primary	73 (66.4)	37 (33.6)***	26 (23.6)	84 (76.4)*	58 (52.7)	52 (47.3)
Secondary	124 (53.7)	107 (46.3)	78 (33.8)	153 (66.2)	105 (45.5)	126 (54.6)
Tertiary	72 (29.7)	170 (70.3)	91 (37.6)	151 (62.4)	145 (59.9)	97 (40.1)
Have health insurance						
Yes	168 (43.9)	215 (56.1)	133 (34.7)	250 (65.3)	213 (55.6)	170 (44.4)
No	101 (50.5)	99 (49.5)	62 (31.0)	138 (69.0)	95 (47.5)	105 (52.5)
Used antibiotics in the past year						
Yes	110 (38.7)	174 (61.3)***	108 (38.0)	176 (62.0)*	166 (58.5)	118 (41.6)**
No	159 (53.2)	140 (46.8)	87 (29.1)	212 (70.9)	142 (47.5)	157,952.5)
Self-rated health						
Fair or poor	51 (54.3)	43 (45.7)	33 (35.1)	61 (64.9)	42 (44.7)	52 (55.3)
Good	183 (45.2)	222 (54.8)	132 (32.6)	273 (67.4)	221 (54.6)	184 (45.4)
Very good	35 (41.7)	49 (58.3)	30 (35.7)	54 (64.3)	45 (53.6)	39 (46.4)
Access to a primary care doctor						
Very easy	49 (35.0)	91 (65.0)**	37 (26.4)	103 (73.6)	90 (64.3)	50 (35.7)**
Easy	173 (48.3)	185 (51.7)	123 (34.4)	235 (65.6)	184 (51.4)	174 (48.6)
Others	47 (55.3)	38 (44.7)	35 (41.2)	50 (58.8)	34 (40.0)	51 (60.0)
Information source about antibiotics						
0	66 (65.4)	35 (34.7)***	32 (31.7)	69 (68.3)	38 (37.6)	63 (62.4)**
1	119 (46.7)	136 (53.3)	81 (31.8)	174 (68.2)	139 (54.5)	116 45.5)
2	84 (37.0)	143 (63.0)	82 (36.1)	145 (63.9)	131 (57.7)	96 (42.3)
Knowledge level of antibiotics						
Low	-	-	85 (31.6)	184 (68.4)	107 (39.8)	162 (60.2)***
High	-	-	110 (35.0)	204 (65.0)	201 (64.0)	113 (36.0)

*p < 0.05; ** *p* < 0.01; *** *p* < 0.001.

Table 4 shows the results of the multivariate analysis concerning knowledge. After controlling for other variables, participants with secondary (aOR = 1.91, 95% CI: 1.12–3.26) and tertiary educational levels (aOR = 5.07, 95% CI: 2.75–9.34) were more likely to have higher levels of antibiotic knowledge than those who had a primary educational level. People who had not used antibiotics in the past year (aOR = 0.65, 95% CI: 0.46–0.93) were likely to have a lower knowledge of antibiotics. Respondents with lower ease of access to a primary care doctor (aOR = 0.58 95% CI: 0.37–0.91; aOR = 0.37, 95% CI: 0.20–0.67) were likely to have a lower level of antibiotic knowledge compared with people with easy access. Furthermore, receiving information from more health professionals was associated with higher knowledge (aOR = 2.07, 95% CI: 1.21–3.55).

**TABLE 4:** Multivariate analysis of factors associated with self-medication of antibiotics and completing antibiotics regimens.

Variable	Knowledge		Self-medication		Completing an antibiotic regimen	
	OR	95% CI	OR	95% CI	OR	95% CI
Residence						
Rural	1.00		1.00		1.00	
Urban	1.11	0.69–1.81	3.40***	1.95–5.93	1.22	0.75–1.98
Age (years)						
18–29	1.00		1.00		1.00	
30–39	1.21	0.74–1.99	1.07	0.65–1.77	1.30	0.80–2.11
40–49	1.59	0.91–2.76	0.84	0.47–1.51	1.02	0.60–1.74
Marital status						
Single	1.00		1.00		1.00	
Married	0.97	0.61–1.55	0.69	0.44–1.10	1.40	0.89–2.20
Educational level						
Primary	1.00		1.00		1.00	
Secondary	1.91*	1.12–3.26	0.90	0.50–1.63	0.70	0.41–1.18
Tertiary	5.07***	2.75–9.34	0.72	0.38–1.37	1.00	0.54–1.84
Have health insurance						
Yes	1.00		1.00		1.00	
No	1.01	0.68–1.49	1.14	0.76–1.71	0.82	0.56–1.20
Used antibiotics in the past year						
Yes	1.00		1.00		1.00	
No	0.65*	0.46–0.93	0.66*	0.45–0.96	0.81	0.57–1.15
Self-rated health						
Fair or poor	1.00		1.00		1.00	
Good	1.44	0.88–2.38	1.09	0.66–1.80	1.41	0.87–2.29
Very good	1.31	0.68–2.54	1.24	0.65–2.39	1.23	0.65–2.32
Access to a primary care doctor						
Very easy	1.00		1.00		1.00	
Easy	0.58*	0.37–0.91	1.43	0.90–2.27	0.71	0.46–1.09
Others	0.37**	0.20–0.67	1.85*	1.00–3.42	0.44**	0.25–0.81
Information source about antibiotics						
0	1.00		1.00		1.00	
1	1.59	0.95–2.67	0.77	0.45–1.32	1.53	0.92–2.55
2	2.07**	1.21–3.55	0.80	0.46–1.40	1.64	0.96–2.80
Knowledge level of antibiotics						
Low	-	-	1.00		1.00	
High	-	-	1.07	0.73–1.58	2.25***	1.56–3.25

*p < 0.05; ** *p* < 0.01; *** *p* < 0.001.

aOR, adjusted odds ratio; CI, confidence interval.

The multivariate analytical results also revealed that participants from urban areas (aOR = 3.40, 95% CI: 1.95–5.93) were more likely to report antibiotic self-medication compared with those from rural areas. Moreover, individuals who had not used antibiotics in the past year (aOR = 0.66, 95% CI: 0.45–0.96) were less likely to report antibiotic self-medication. Respondents with a lower ease of access to a primary care doctor (aOR = 1.85, 95% CI: 1.00–3.42) were likely to report antibiotic self-medication compared with people with easy access.

Table 4 further reveals that having difficulty accessing a primary care doctor (aOR = 0.44, 95% CI: 0.25–0.81) was associated with a reduced likelihood of completing the full course of antibiotics. Additionally, having a high level of antibiotic knowledge (aOR = 2.25, 95% CI: 1.56–3.25) was associated with an increased likelihood of completing the full course of antibiotic medication. Furthermore, we analyzed the data of 284 respondents who had taken antibiotics in the previous year to understand their status of completing antibiotic courses . The results were stable, and are shown in Supplementary Table S1.

## 4 Discussion

Easy and unregulated access to antibiotics in Indonesia, coupled with poor knowledge of antibiotic usage, is a combination that will likely lead to inappropriate use and worsen the antibiotic-resistance problem.

This is the first study in Malang, Indonesia to assess the knowledge and practices of women in a primary healthcare setting. This study demonstrated that women with higher education, more sources of antibiotic information, previous antibiotic experience, and easy access to primary care physicians scored higher in antibiotic knowledge. However, living in urban areas or using antibiotics in the past year may have made them prone to self-medication. Further, difficulty accessing a primary care doctor and low antibiotic knowledge may cause them to not complete antibiotic regimens.

### 4.1 Factors associated with antibiotic knowledge

Consistent with studies conducted in Italy and Sweden (Napolitano et al., 2013; Vallin et al., 2016), higher educational attainment was associated with a higher level of antibiotic knowledge. The more educated are more likely to obtain relevant knowledge through the education system and to have better understanding of the terminologies used for antibiotic usage. However, conflicting results have been reported. Authors assessed respondents’ knowledge of bacterial resistance to antibiotics and reported that more educated respondents were less knowledgeable about bacterial resistance in Yemen (Belkina et al., 2014). A randomized survey found that educational attainment was not significantly associated with a lower knowledge of antibiotics (Jamhour et al., 2017). In summary, younger people and those with high educational levels have greater levels of antibiotic knowledge in most countries. However, Belkina et al. reported lower antibiotic knowledge among people with higher educational levels, as less educated people would consult their physicians beforehand for fear of side effects (Belkina et al., 2014). Therefore, their knowledge of antibiotic use would increase. Moreover, their study had 40.2% male respondents, with 18.3% respondents aged below 20. Our study’s population was female, concentrated in the age range of 18–29, with their higher education levels significantly associated with higher levels of antibiotic knowledge. The aforementioned factors may explain the conflicting results, and they highlight the importance of focusing on female subjects to promote antibiotic knowledge education.

This study found that people with experience of antibiotic use performed better in terms of antibiotic knowledge. Consistent with Salm et al.’s findings, respondents who had received antimicrobial treatment in the past 12 months had more correct responses regarding antibiotic knowledge (Salm et al., 2018). Another study suggested that antibiotic-experienced patients may have gained more knowledge because of their recent engagement with the subject of antibiotic use (Shebehe et al., 2021).

We found that women who had easy access to primary care physicians had higher antibiotic knowledge. According to a previous study, those who reported that they had access to a formal health care provider were almost three times as likely to have adequate health literacy compared with those without formal health care provider access (Dunn-Navarra et al., 2012). Additionally, another study’s findings suggest that interventions by health care professionals can help improve knowledge on antibiotics (Pham-Duc and Sriparamananthan, 2021).

### 4.2 Factors associated with antibiotic self-medication

Our results showed that living in an urban area is associated with antibiotic self-medication. Urban areas may have greater access to antibiotics than rural areas; considering multiple outlets such as pharmacies, general stores, friends, relatives, and kiosks (Hadi et al., 2008), urban dwellers are more likely to use antibiotics without a prescription. Conflicting results have been reported in the literature. Hidayah et al. highlighted that people with high socioeconomic status, higher education, living in urban areas, higher income, and women may have better knowledge, attitudes, and practices regarding antibiotic use (Karuniawati et al., 2021). This potential explanation could be supported by the higher proportion of inappropriate prescriptions in rural areas from a narrative review of antimicrobial stewardship (Yau et al., 2021). The challenges faced by primary health care providers in rural and remote areas of Indonesia include limited resource allocation and a lower number of pharmacies than in urban areas, which may result in people being more likely to obtain antibiotics on their own.

Furthermore, a Southern European study reported that a lack of proper antibiotic use knowledge was associated with a higher risk of self-medication (Grigoryan et al., 2008; Papaioannidou et al., 2009). Self-medication was higher among patients with a good knowledge of antibiotics (McNulty et al., 2007; Pavydė et al., 2015). However, in our study, antibiotic knowledge was not associated with self-medication.

Our study found that individuals who had not used antibiotics in the past year (aOR = 0.66, 95% CI: 0.45–0.96) were less likely to report antibiotic self-medication. Some studies reported that previous experience related to the efficacy of antibiotic treatments may be a reason for self-medication (Al-Azzam et al., 2007; Mainous et al., 2008). A study conducted in South Carolina, United States showed that self-medication with antibiotics was driven by past experience with antibiotics.

For the first time of antibiotic use, patients usually seek medical care from care workers such as pharmacists. When they re-experience symptoms that they have experienced before, they may seek treatment that had been successful for them in the past (Mainous et al., 2008). Furthermore, a systematic review of antimicrobial self-medication indicated that past successful use may be a determining factor associated with self-medication (Chowdhury et al., 2009; Ocan et al., 2015). Due to the negative influence of antibiotics on self-medication, it is necessary to encourage local residents to seek medical assistance instead of self-medicating with antibiotics.

### 4.3 Factors associated with completion of antibiotic regimen

Our findings indicate that half of the participants did not complete antibiotic regimens. Consistent with a previous study, 45% of respondents expressed a negative attitude regarding stopping antibiotic use before completing the course once they felt better (Awad and Aboud, 2015). This misconception may lead to more serious antibiotics resistance. Therefore, we also explored potential factors that may be associated with participants’ attitude toward completing antibiotic regimens.

Our findings suggest that completion of the antibiotic regimen was associated with a higher knowledge level and more information sources. A study in Lithuania indicated that two-thirds of the participants had insufficient knowledge regarding antibiotics, which may lead to increased non-adherence and self-medication (Pavydė et al., 2015).

Previous studies have showed that adherence to antibiotic therapy and appropriate antibiotic use are strongly associated with better public awareness and knowledge of antibiotics (McNulty et al., 2007; You et al., 2008; Chan et al., 2012; Awad and Aboud, 2015). Regarding the completion of an antibiotic course, having difficulty accessing a primary care doctor was associated with a reduced likelihood of completing antibiotic courses. Improving access to primary care doctors is an important step toward achieving proper antibiotic usage. Additionally, obtaining knowledge from health care professionals and having a high level of knowledge regarding antibiotics reinforce the completion of antibiotic courses, thus highlighting that knowledge, as well as the source of that knowledge, are precursors for proper antibiotics use (Ganguly et al., 2011; Han et al., 2020).

### 4.4 Relevant description of attitude toward antibiotics use

The authors included the statement, “When I get a cold, I take antibiotics to help me get better more quickly” to determine the attitude scores of antibiotic use (Mouhieddine et al., 2015). According to our results, participants had a lower correct response rate to the items “Antibiotics work on most common colds” and “Antibiotics are effective against viruses.” This revealed that participants may have a misunderstanding attitude toward antibiotic use. A potential explanation is confusion regarding the term “germs,” which is generally used during medical counseling rather than microbiological terms such as “bacteria” and “virus.” This misconception may affect participants’ subsequent behaviors, such as expecting doctors to prescribe an antibiotic for common cold and putting pressure on the doctor to meet patients’ expectations (Mouhieddine et al., 2015).

This was a cross-sectional study that utilized convenience sampling; hence, causal inferences cannot be drawn and the results’ generalizability is limited. Convenience sampling may have led to selection bias. Other potential confounding factors such as financial status, details concerning medication reimbursement, attitudinal variables, and the frequency of antibiotic-use were not collected, and thus, our results should be interpreted with caution.

Several studies have suggested that women have higher knowledge levels and better compliance to complete treatment (Fatokun, 2014; Karuniawati et al., 2021; Endashaw Hareru et al., 2022). Furthermore, they are more willing to contact health care providers. (Baker et al., 2007; Thompson et al., 2016; Burnside et al., 2018; Shebehe et al., 2021), and may be prioritized in campaign programs. Furthermore, most women are the primary caregivers of family members (Togoobaatar et al., 2010). Thus, they are more influential.

While establishing a tailor-made national antibiotic communication strategy, it is essential to understand the levels of knowledge regarding antibiotics and potential factors associated with antibiotic knowledge, completion of antibiotic regimens, and practicing self-medication in women.

The behavior of buying antibiotics not included in the OWA list without prescription still exists. The drivers of inappropriate antibiotic dispensing included poor knowledge of antibiotics and AMR, incentives to elevate medicine sales, and insufficient regulatory enforcement (Ferdiana et al., 2021). In Indonesia, the general population is unaware regarding OWA, making it difficult for them to differentiate which antibiotics require prescriptions. Therefore, the regulation at the sales end should be strengthened to promote appropriate antibiotics use.

## 5 Conclusion

The key findings of our study will help policymakers in Indonesia to establish future interventions for women to improve their antibiotic use.

More stringent regulation of the dissemination of antibiotics is the first step in combating their misuse. If Policymakers tend to reduce inappropriate antibiotic use in women. Priority advocates are recommended for urban residents who have experience of antibiotic use in the previous year. Additionally, increasing public awareness and knowledge is essential for changing antibiotic usage behaviors. There seem to be knowledge gaps in women in terms of the effectiveness of antibiotics against different types of pathogens and activities, which can compromise antibiotic effectiveness. It is therefore important to increase their awareness, particularly regarding diseases against which antibiotics are effective, and activities such as unnecessary use of antibiotics in healthy animals may affect their overall effectiveness among humans. More communication channels should be included in the overall scheme to improve public awareness regarding antibiotics and accessibility of health professionals.

## Data Availability

The original contributions presented in the study are included in the article/Supplementary Materials, further inquiries can be directed to the corresponding author.
