# Supplementary material for: Knowledge and practices related to antibiotic use among women in Malang, Indonesia
Source: Front Pharmacol. 2022 Oct 24;13:1019303. doi: 10.3389/fphar.2022.1019303 (PMC9637850; doi:10.3389/fphar.2022.1019303)
Supplement: Supplementary file 1 [file Table1.DOCX]

| **Supplementary Table S1 Factors associated with completing antibiotics regimens for the 284 participants who took antibiotics in previous year** | | | | | |
| --- | --- | --- | --- | --- | --- |
| **Variable** | **Completing an antibiotic regimen** | | | |  |
|  | **OR** | **95% CI** | **aOR** | **95% CI** |  |
| Residence |  |  |  |  |  |
| Rural | 1.00 |  | 1.00 |  |  |
| Urban | 1.06 | 0.61~1.84 | 0.63 | 0.30~1.34 |  |
| Age (years) |  |  |  |  |  |
| 18~29 | 1.00 |  | 1.00 |  |  |
| 30~39 | 1.92* | 1.05~3.51 | 2.41* | 1.17~4.96 |  |
| 40~49 | 0.92 | 0.50~1.70 | 1.14 | 0.53~2.46 |  |
| Marital status |  |  |  |  |  |
| Single | 1.00 |  | 1.00 |  |  |
| Married | 1.05 | 0.65~1.70 | 0.87 | 0.45~1.67 |  |
| Educational level |  |  |  |  |  |
| Primary | 1.00 |  | 1.00 |  |  |
| Secondary | 1.05 | 0.53~2.10 | 1.35 | 0.61~2.98 |  |
| Tertiary | 2.13* | 1.08~4.20 | 2.97* | 1.25~7.08 |  |
| Have health insurance |  |  |  |  |  |
| Yes | 1.00 |  | 1.00 |  |  |
| No | 0.90 | 0.53~1.53 | 0.90 | 0.49~1.67 |  |
| Self-rated health |  |  |  |  |  |
| Fair or poor | 1.00 |  | 1.00 |  |  |
| Good | 1.22 | 0.63~2.34 | 1.05 | 0.51~2.12 |  |
| Very good | 1.33 | 0.56~3.18 | 1.05 | 0.40~2.71 |  |
| Access to a primary care doctor |  |  |  |  |  |
| Very easy | 1.00 |  | 1.00 |  |  |
| Easy | 0.44** | 0.24~0.79 | 0.44* | 0.23~0.85 |  |
| Others | 0.48 | 0.21~1.09 | 0.50 | 0.20~1.20 |  |
| Information source about antibiotics |  |  |  |  |  |
| 0 | 1.00 |  | 1.00 |  |  |
| 1 | 1.53 | 0.71~3.31 | 1.39 | 0.61~3.15 |  |
| 2 | 1.87 | 0.86~4.05 | 1.64 | 0.71~3.80 |  |
| Knowledge level of antibiotics |  |  |  |  |  |
| Low | 1.00 |  | 1.00 |  |  |
| High | 1.83* | 1.13~2.98 | 1.43 | 0.83~2.45 |  |
|  |  |  |  |  |  |

* *p* < 0.05; ** *p* < 0.01; *** *p* < 0.001.

OR, odds ratio; CI, confidence interval.
